# A Case Report of Direct‐Acting Antiviral Therapy for Chronic Hepatitis C in a Patient With Dementia Treated in Collaboration With Multiple Specialists

**DOI:** 10.1155/crhe/5540732

**Published:** 2026-01-27

**Authors:** Tomoki Tanie, Kotaro Kanda, Hirotoshi Fujikawa

**Affiliations:** ^1^ Department of General Medicine, Japan Community Healthcare Organization Yokohama Central Hospital, Kanagawa, Japan

## Abstract

A 77‐year‐old female with dementia was transferred from her family clinic to our hospital with a 2‐day history of appetite loss and was diagnosed with pneumonia and urinary tract infection upon admission. Laboratory investigation revealed hepatitis C virus antibody positivity and an elevated hepatitis C virus ribonucleic acid level of 3.6 Log IU/mL; therefore, direct‐acting antiviral therapy was initiated. Although the patient requested treatment for hepatitis C, managing her medication was difficult because of dementia, as she lived alone and had no family, which required her to take medication under supervision, even on holidays. After discharge, the patient was treated with glecaprevir hydrate and pibrentasvir for 8 weeks by a hepatologist with biweekly visits to monitor adverse events. The hepatitis C virus ribonucleic acid test result was negative after 4 weeks of treatment, and we asked her family physician to confirm a sustained virologic response. Collaboration among multiple specialists, both within and outside the hospital, is essential for facilitating the treatment of such patients.

## 1. Introduction

Hepatitis C virus (HCV) infection is a major cause of cirrhosis and hepatocellular carcinoma throughout the world [1]. HCV is bloodborne, and most infections are acquired through unsafe healthcare procedures or injection practices [2].

Chronic hepatitis C is defined as the persistence of HCV ribonucleic acid (RNA) in the blood for more than 6 months after the onset of acute infection. Approximately 55%–85% of patients with acute hepatitis C develop chronic hepatitis C, which can subsequently lead to progressive fibrosis, cirrhosis, end‐stage liver disease, and hepatocellular carcinoma [3]. Recently, direct antiviral activity (DAA) oral drugs, characterized by high cure rates over short treatment durations and good tolerability, have transformed chronic hepatitis C into a curable disease, demonstrating extraordinary clinical efficacy. Substantial price reductions and expansion of access in resource‐limited settings have provided a new impetus for the control and elimination of hepatitis C [4].

Herein, we report the case of a patient with chronic hepatitis C, for whom DAA treatment was initially considered difficult due to dementia; however, successful treatment was achieved through collaboration with multiple specialists.

## 2. Case Presentation

A 77‐year‐old woman presented to the emergency department with a 2‐day history of appetite loss. A qualitative urine test performed at her family doctor’s clinic revealed turbidity and white blood cells. Accordingly, the doctor transported the patient to our hospital with a suspected urinary tract infection. Her medical history included hypertension and dementia, both of which were diagnosed at a family doctor’s clinic. She reported no tobacco or illicit drug use, alcohol consumption, known allergies, or family history of hereditary diseases, and was not taking any medications.

Upon arrival at our hospital, her vital signs were as follows: Glasgow Coma Scale, E4V4M6; body temperature, 36.5°C; blood pressure, 117/86 mmHg; pulse rate, 90/min; respiratory rate, 16/min; and SpO_2_, 97% on room air. Physical examination revealed a prompt pupillary light reflex, with normal respiratory and cardiac sounds. The abdomen showed no tenderness; however, costovertebral angle tenderness was observed. Leg edema was not observed, and laboratory examination revealed an elevated white cell count of 15,100/μL (reference range: 3200–9000/μL) and C‐reactive protein level of 9.23 mg/dL (reference range: 0.00–0.30 mg/dL). A qualitative urine test revealed turbidity and white blood cells, which could not be counted by the analyzer because of excessive sample turbidity (Table 1).

**Table TABLE 1:** Laboratory examination results on admission.

*Blood cell count*	
WBC (/μL)	15,100
RBC (/μL)	368 × 10^4^
Hb (g/dL)	11.1
Hct (%)	34.7%
Plt (/μL)	30.8 × 10^4^
MCV (fL)	94.5
MCH (pg)	30.2

*Urine test*	
Cloudiness	3+
pH	7.5
Nitrites	—
Protein	4+
Ketone body	—
Glucose	3+

*Biochemical test*	
TP (g/dL)	7.4
Alb (g/dL)	2.7
BUN (g/dL)	23.6
Cre (mg/dL)	1.09
eGFR (mL/min/1.73 m^2^)	37.5
AST (U/L)	13
ALT (U/L)	8
γ‐GTP (U/L)	14
AL‐P (U/L)	101
CK (U/L)	56
T‐Bil (mg/dL)	0.7
Na (mEq/L)	136
K (mEq/L)	3.8
Cl (mEq/L)	104
Glucose (mg/dL)	123
CRP (mg/dL)	9.23
HBs‐Ag (IU/mL)	0.02
HCV‐Ab	14.78

*Note:* Hb, hemoglobin; Hct, hematocrit; Alb, albumin; Cre, creatinine; AST, aspartate aminotransferase; ALT, alanine transferase; γ‐GTP, γ‐glutamyl transferase; AL‐P, alkaline phosphatase; T‐Bil, total bilirubin; Na, sodium; K, potassium; Cl, chloride; HBs‐Ag, hepatitis B surface antigen; HCV‐Ab, hepatitis C virus antibody.

Abbreviations: BUN, blood urea nitrogen; CK, creatine kinase; CRP, C‐reactive protein; eGFR, estimated glomerular filtration rate; HPF, high power field; MCH, mean corpuscular hemoglobin; MCV, mean corpuscular volume; RBC, red blood cell; TP, total protein; WBC, white blood cell.

Pharyngeal swab antigen tests for Severe acute respiratory syndrome coronavirus 2 and influenza A and B viruses yielded negative results. Electrocardiography revealed a normal sinus rhythm, and chest radiography showed no cardiomegaly (cardiothoracic ratio: 40.8%); however, reticular shadows were observed in the left lower lung field (Figure 1(a)). Chest computed tomography (CT) revealed reticular shadows in both lower lung lobes (Figure 1(b)), and abdominal CT revealed no hepatic masses (Figure 1(c)).

Figure FIGURE 1Chest radiography and computed tomography (CT) findings upon admission. (a) Presence of reticular shadows in the left lower lobes of the lung field. (b) Presence of reticular shadows in both the lower lobes of the lung field. (c) No hepatic mass was observed.

**Figure:**
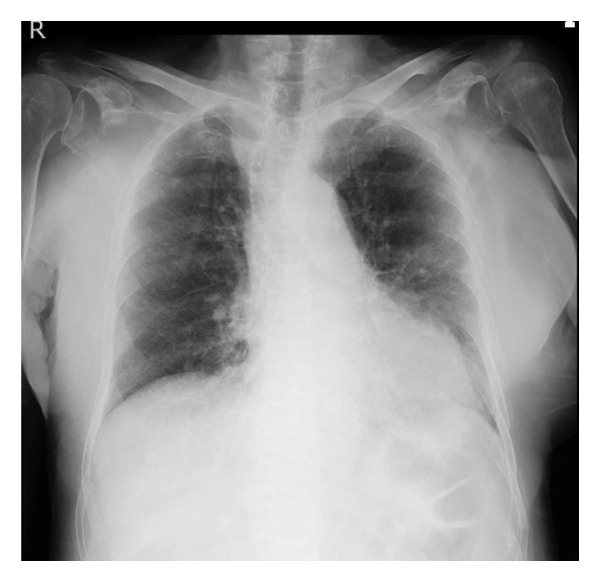
(a)

**Figure:**
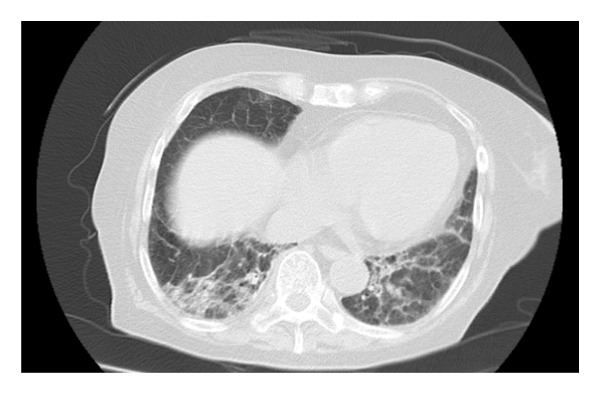
(b)

**Figure:**
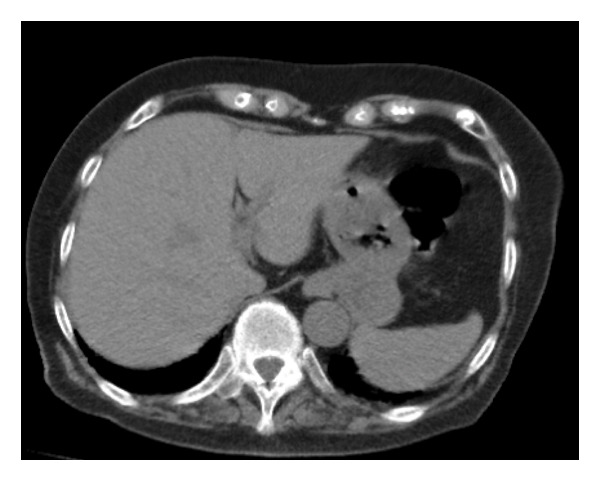
(c)

The patient was admitted with pneumonia and urinary tract infection and received ampicillin sodium (6 g/day) for 7 days, after which her appetite improved. A few days later, urine culture results showed *Proteus mirabilis* infection, which is sensitive to ampicillin sodium. Laboratory examination for viral hepatitis, performed as part of nosocomial infection control, revealed a positive HCV antibody of 14.78. Based on these results, HCV RNA was measured and showed an elevated level of 3.6 Log IU/mL (reference range: negative) and genotyping revealed Genotype 2. Furthermore, abdominal ultrasonography revealed a 7‐mm liver cyst in Segment VIII (Figure 2), and FibroScan revealed liver stiffness of 7.5 kPa, indicating middle‐range stiffness.

**Figure FIGURE 2:**
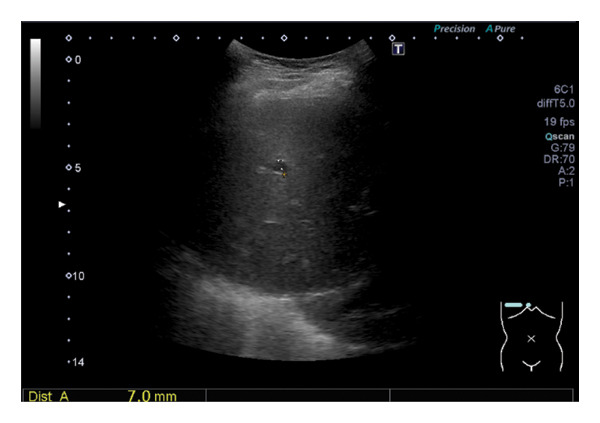
Abdominal ultrasonography findings during hospitalization. Presence of a 7 mm liver cyst in Segment VIII.

After confirming the diagnosis of chronic hepatitis C, we informed the patient and explained that she was at risk of developing cirrhosis and hepatocellular carcinoma in the future. She understood and requested therapy for chronic hepatitis C. Although DAA therapy was initiated, it was difficult for her to manage her medication because of dementia. She lived alone and had no family; therefore, her home environment had to be modified. Discussions were held with her primary care physician, hepatologist, and care manager, who prepared the care plan services for older adults requiring support and coordinated with local authorities and service providers. To ensure daily adherence to the DAA therapy, a helper visited her home on Saturdays and Sundays, while the care manager coordinated social services during her hospitalization. The patient was discharged on Day 45 in a stable condition.

After discharge, the patient was treated with glecaprevir hydrate and pibrentasvir for 8 weeks by a hepatologist, with biweekly visits to monitor for adverse events and ensure that no renal or hepatic impairment occurred (Table 2). The implementation of social services planned by the care manager enabled the completion of DAA therapy, and HCV RNA was negative after 4 weeks of treatment. In principle, HCV RNA negativity at 12 weeks posttreatment, referred to as a sustained virological response (SVR), should be confirmed. However, the patient refused to visit our hospital; therefore, we asked her family doctor to monitor for SVR.

**Table TABLE 2:** Laboratory examinations after discharge from the hospital.

	Day 62	Day 76	Day 90	Day 104	Day 118
WBC (/μL)	8430	8250	8660	8150	8940
Hb (g/dL)	11.0	11.8	12.0	11.3	11.3
Plt (/μL)	21.5 × 10^4^	33.3 × 10^4^	25.8 × 10^4^	29.6 × 10^4^	27.1 × 10^4^
TP (g/dL)	7.1	7.8	7.3	7.1	7.1
Alb (g/dL)	3.2	3.6	3.3	3.2	3.3
BUN (mg/dL)	14.9	16.3	17.7	18.7	16.1
Cre (mg/dL)	0.88	1.12	1.01	1.06	1.02
eGFR (mL/min/1.73 m^2^)	47.4	36.4	40.8	38.7	40.3
CK (U/L)	52	66	55	49	55
AST (U/L)	32	18	14	12	11
ALT (U/L)	26	10	6	5	5
γ‐GTP (U/L)	16	14	11	10	9
AL‐P (U/L)	107	170	196	199	199
T‐Bil (mg/dL)	0.3	2.0	1.1	1.2	1.5
Na (mEq/L)	141	140	141	140	141
K (mEq/L)	3.9	4.8	4.2	4.4	3.8
Cl (mEq/L)	106	106	107	103	107

*Note:* Hb, hemoglobin; Plt, platelet; Alb, albumin; Cre, creatinine; AST, aspartate aminotransferase; ALT, alanine transferase; AL‐P, alkaline phosphatase; γ‐GTP, γ‐glutamyl transpeptidase; T‐Bil, total bilirubin; Na, sodium; K, potassium; Cl, chloride.

Abbreviations: BUN, blood urea nitrogen; CK, creatine kinase; eGFR, estimated glomerular filtration rate; TP, total protein; WBC, white blood cell.

## 3. Discussion

Improving medication adherence in patients with dementia is a serious concern. Factors associated with improved adherence include communication between physicians and patients, mutual agreement on what is helpful, shared responsibilities, and patient self‐management [5]. However, deficits in cognitive processes due to dementia predispose older adults to medication nonadherence by impairing their ability to plan, organize, and execute medication management tasks. Environmental and systemic factors modulate medication adherence [6–8]. Patients with chronic hepatitis C have better treatment experiences, higher adherence to treatment, and substantial improvements in health‐related quality of life during treatment with DAAs [9]. Therefore, it is important to create an environment in which patients with chronic hepatitis C can continue treatment with DAAs even in the presence of dementia.

In this case, the patient had not taken any medications for a long time, and resuming treatment may have been a new experience. Owing to the difficulty of adhering to medication on her own, the use of a healthcare system was essential.

Generally, navigating the healthcare system for patients with dementia is challenging and usually involves caregivers [10]. In the present case, the patient had no family or kin, which made it difficult for her to access the healthcare system. In response, we propose strengthening the collaboration with multiple specialists, both inside and outside the hospital. Primary care practices play an important role in coordinating the multidisciplinary management of patients to ensure comprehensive care [11].

One clinical trial analysis to determine the positive predictive value (PPV) of SVR at Posttreatment week 4 (SVR4) for achieving SVR12 in patients with HCV without cirrhosis or with compensated cirrhosis receiving glecaprevir hydrate and pibrentasvir reported that regardless of treatment duration, the PPV of SVR4 for SVR12 was > 99% in both groups, and not achieving SVR4 had a 100% negative predictive value and sensitivity for all groups. Therefore, SVR4 was highly predictive of SVR12 in patients with HCV [12]. A previous prospective observational study on the efficacy and safety of DAA therapy in patients with cancer and chronic HCV infection reported that 8 weeks of DAA therapy was highly effective and safe for patients with HCV infection [13]. Unfortunately, we could not recognize SVR12 of HCV owing to the worsening of dementia, but SVR4 negativity strongly predicted treatment success with glecaprevir/pibrentasvir in this case.

Nonetheless, achieving HCV RNA–negativity with support from the healthcare system is a valuable outcome for the patient.

## 4. Conclusions

Multidisciplinary collaboration is essential to improve medication adherence in patients with dementia. In the present case, multidisciplinary collaboration from inside and outside the hospital was key to the successful treatment of hepatitis C.

## Author Contributions

T.T., K.K., and H.F. contributed substantially to the conception or design of the work.

## Funding

No specific funding was received from any organization in the public, commercial, or not‐for‐profit sectors to conduct the work described in this article.

## Disclosure

All authors approved the submitted version and agree to be responsible for all aspects of the work in ensuring that any questions relating to the accuracy or completeness of any part of the work are properly investigated and resolved.

## Ethics Statement

In our institution, case reports do not require approval from the ethics committee for publication. Therefore, institutional approval was not necessary for publishing the details of this case.

## Consent

Informed consent was obtained from the patient by the corresponding author. Details of the patient have been anonymized as much as possible.

## Conflicts of Interest

The authors declare no conflicts of interest.

## Data Availability

Data are available on request due to privacy/ethical restrictions.
